# Application of Band-Selective HSQC NMR in Species Discrimination and Adulteration Identification of *Panax Linn*

**DOI:** 10.3390/molecules28114332

**Published:** 2023-05-25

**Authors:** Congcong Guo, Jiyang Dong, Lingli Deng, Kiankai Cheng, Yue Xu, Haowen Zhu, Anjun Deng, Xia Zhou, Hailin Qin, Yinghong Wang

**Affiliations:** 1 Institute of Meteria Medica, Chinese Academy of Medical Sciences & Peking Union Medical College, Beijing 100050, China; guocongcong@imm.ac.cn (C.G.);; 2 Department of Electronic Science, Fujian Provincial Key Laboratory for Plasma and Magnetic Resonance, Xiamen University, Xiamen 361005, China; jydong@xmu.edu.cn; 3 Department of Information Engineering, East China University of Technology, Nanchang 330013, China; 4 Innovation Centre in Agritechnology, Universiti Teknologi Malaysia, Pagoh 84600, Johor, Malaysia

**Keywords:** identification of *Panax Linn*, adulteration identification, heteronuclear single quantum coherence, selective excitation

## Abstract

The quality of *Panax Linn* products available in the market is threatened by adulteration with different *Panax* species, such as *Panax quinquefolium* (*PQ*), *Panax ginseng* (*PG*), and *Panax notoginseng* (*PN*). In this paper, we established a 2D band-selective heteronuclear single quantum coherence (bs-HSQC) NMR method to discriminate species and detect adulteration of *Panax Linn*. The method involves selective excitation of the anomeric carbon resonance region of saponins and non-uniform sampling (NUS) to obtain high-resolution spectra in less than 10 min. The combined strategy overcomes the signal overlap limitation in ^1^H NMR and the long acquisition time in traditional HSQC. The present results showed that twelve well-separated resonance peaks can be assigned in the bs-HSQC spectra, which are of high resolution, good repeatability, and precision. Notably, the identification accuracy of species was found to be 100% for all tests conducted in the present study. Furthermore, in combination with multivariate statistical methods, the proposed method can effectively determine the composition proportion of adulterants (from 10% to 90%). Based on the PLS-DA models, the identification accuracy was greater than 80% when composition proportion of adulterants was 10%. Thus, the proposed method may provide a fast, practical, and effective analysis technique for food quality control or authenticity identification.

## 1. Introduction

*Panax quinquefolium* (*PQ*), *Panax ginseng* (*PG*), and *Panax notoginseng* (*PN*) are valuable traditional Chinese medicines. They are among the most common and main commercial *Panax Linn* of the Araliaceae family, with vast and different medicinal properties. For example, *PG* and *PQ* were found to regulate nervous system, *PG* is known to have excitatory effect, and *PQ* is reported to have sedative effect [1]. In addition, *PN* is well known to have beneficial effects on the cardiovascular system and may promote blood circulation and remove blood stasis. Due to their high value, adulteration of higher-priced species with other lower-priced ones is not uncommon in the market. Therefore, there is a need for a quick and effective method for quality control of *Panax* products to protect the interests of consumers.

In recent years, some efforts have been made to determine *Panax Linn*, including investigation through DNA sequencing [2,3], mass spectrometry (MS) [4,5,6] and chromatography [7,8]. DNA sequencing can identify minor genetic differences among subspecies [9]. MS and chromatography focus on ginsenosides due to their different composition in *Panax Linn*. Using the feature, Wan, J. B identified *PQ, PG*, and *PN* effectively by characteristic maps [10]. These methods still suffer from the challenge of poor repeatability and robustness.

Nuclear magnetic resonance (NMR) is a non-destructive analytical technique that can be used to investigate the structure of organic compounds. NMR analysis offers high reproducibility, high throughput, and simple sample preparation. It is a valuable qualitative and quantitative analysis technology in various fields, including quality control of food [11,12,13,14], medicine [15,16,17], polymer [18,19,20], and others [21,22]. ^1^H NMR is the most widely used technology due to its speed and simplicity [11,15,16,18]. However, complex mixtures often suffer from severe peak overlapping, which can be addressed by using 2D NMR techniques such as heteronuclear single quantum coherence (HSQC). In HSQC, the indirect dimension (f1 domain) is the ^13^C spectrum (140 ppm), which has display space more extensive than that of ^1^H nucleus (20 ppm). Thus, it can effectively separate the overlapped signals in ^1^H NMR. Previous studies [17,19,23,24] had indicated that 2D qNMR is suitable for complex mixtures, but the resolution of the f1 domain in HSQC is often limited by the number of free induction decay (FID) experiments required, which may result in a long collection time.

To address this issue, we consider two methods which may reduce the acquisition time for a high resolution 2D NMR analysis of *Panax* species. Firstly, band-selective (bs-) HSQC can restrict the f1 dimension by selectively exciting ^13^C region of interest to collect high-resolution spectra [25,26]. Secondly, non-uniform sampling (NUS) uses specific algorithms to obtain high-resolution spectra from short data records [27,28]. Together, we combined 2D bs-HSQC with NUS to analyze compounds in *Panax* species to improve the practicability of 2D NMR in the field of its quality control.

In the present study, we used the combined method to identify *PQ*, *PG*, and *PN* species and their mutual adulterations. The composition of ginsenosides varies significantly in *Panax Linn*; thus, comprehensive elucidation is essential for quality control. Therefore, the work utilized saponins extract as subject to collect bs-HSQC spectra. The obtained cross-peak integration was further studied by multivariate statistical analysis to identify *PQ*, *PG*, and *PN* species and their adulteration. The study provided a fast, practical, and effective NMR analysis method for the identification and authenticity detection of *Panax Linn*, which may be used as a reference for the analysis of other plant materials.

## 2. Results and Discussion

### 2.1. The Establishment of Bs-HSQC NMR Coupled with NUS for Ginsenosides

**Figure 1:**
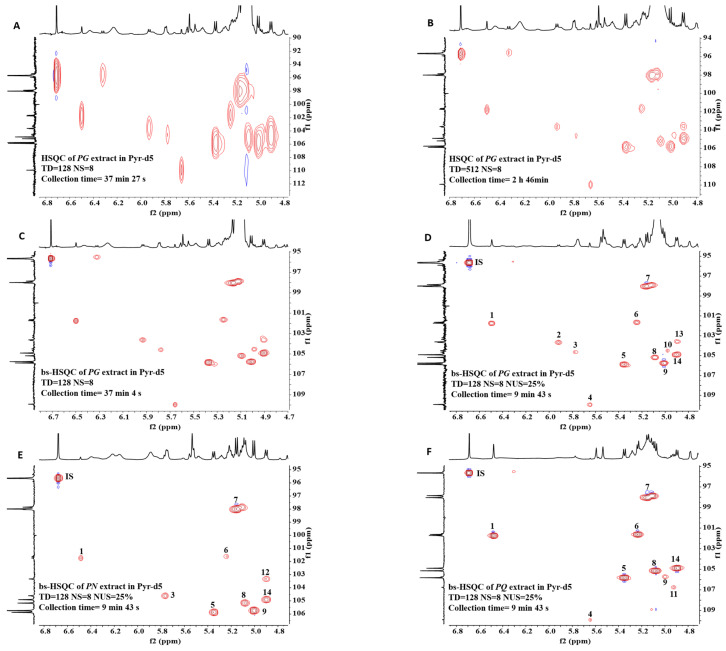
Comparison of HSQC and bs-HSQC NMR (500 MHz, Pyr-d5) spectra. All spectra had a NS (number of scans) of 8. The TD of all bs-HSQC was 128. (**A**) HSQC without NUS of *PG* extract. TD = 128, acquisition time was 37 min 27 s. (**B**) HSQC without NUS of *PG* extract. TD = 512, acquisition time was 2 h 46 min. (**C**) Bs-HSQC without NUS of *PG* extract. TD = 128, acquisition time was 37 min 7 s. The results showed that bs-HSQC produced high-resolution spectra in a shorter time. (**D**) Bs-HSQC with 25% NUS of *PG* extract. (**E**) Bs-HSQC with 25% NUS of *PN* extract. (**F**) Bs-HSQC with 25% NUS of *PQ* extract. Introducing 25% NUS reduced the acquisition time to 9 min 43 s. The numbers in the spectra (**D**–**F**) correspond to No. in Table 1, indicating peak attribution.

Ginsenosides are a group of glycosylated triterpenes with diversity derivatives, and they have characteristic resonance signals. The anomeric ^1^H peaks of sugars usually appear between 4 and 6 ppm, but the signal overlapping in this region is severe, which makes it challenging to obtain accurate integration (f2 dimension in Figure 1A). As 2D HSQC can expand overlapping signals in the carbon dimension, it was used to analyze the extract. However, the spectral width of HSQC in carbon dimension is so wide—usually about 140 ppm—which requires more FIDs in the carbon dimension to achieve a high-resolution spectrum. As a result, the experimental efficiency was very low, with the collection time being 2 h 46 min (Figure 1B). To address this issue, we used bs-HSQC to selectively excite only the interested carbon regions, reducing the spectral width in the carbon dimension to shorten the experimental time by recording fewer FIDs. The anomeric ^13^C peaks of sugars are typically between 95 and 110 ppm, making bs-HSQC ideal for selectively exciting the carbon regions. A high-resolution spectrum can be obtained within 40 min (Figure 1C).

**Figure 2:**
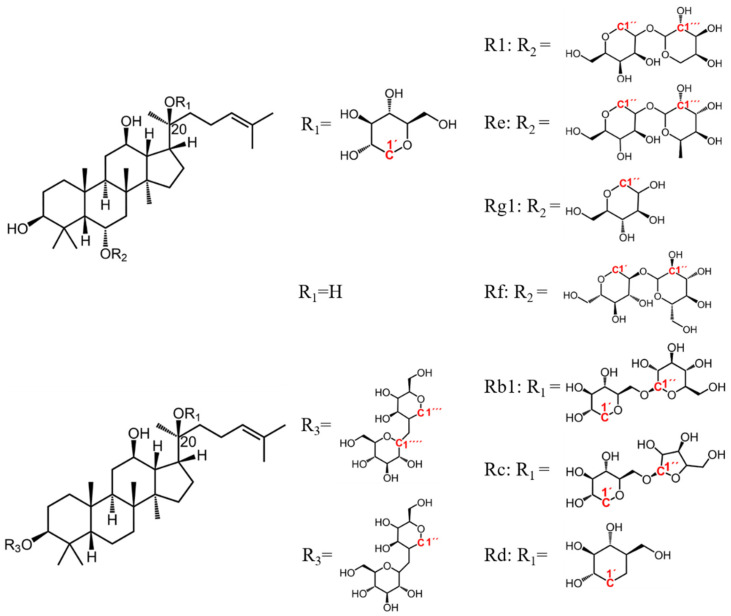
Main ginsenosides in *Panax Linn*. The attributed anomeric carbons were highlighted in red.

Among the ginsenosides isolated from *Panax Linn*, Rb1, Rc, Rd, Re, Rf, and Rg1 (Figure 2) typically constitute more than 90% and are usually regarded as the major ginsenosides [29], with characteristic R1 in *PN*. We spiked bs-HSQC of the extract with ginsenosides standards and identified 12 peaks (Figure 1D–F). The results and their existence in medicinal materials were shown in Table 1.

Absolute qHSQC has more rigorous requirements for acquisition parameters and time. To achieve absolute quantification, the relaxation delay between two FIDs (D1 in Bruker) must be five times the longitudinal relaxation time (*T1*) to ensure the complete relaxation of the target groups. Moreover, heteronuclear coupling constant (*J_CH_*) is another crucial factor that cannot be ignored [20]. Thus, absolute quantification based on conventional HSQC is not practical for quality control purposes. Instead, we utilized the obtained anomeric cross-peak integration as the variable for multivariate statistical analysis to achieve relative quantification, where it is not necessary to set D1 to 5*T1*, and other factors affecting signal intensity can be neglected. To ensure the stability of the target signal integral value, the number of scans (NS) must be optimized to obtain sufficient signal to noise ratio (SNR). We collected spectra from six samples of each species using NS of 4, 8, 12 and 16, and analyzed their corresponding average SNR. Based on the result, NS was set to 8 to balance SNR and experimental time. Although the SNR of R1-1‴ (δ 104.55/5.78) in *PG* was close to limit of quantitation (LOQ) of 10, the influence of its integration error can be neglected, because it was not found present in *PQ* and has a high content in *PN* with the SNR of 38 (Table S1).

In the present study, NUS was used to reduce experimental time, but a previous report showed that NUS may reduce signal intensity of small peaks [30]. Therefore, we took six samples from *PQ*, *PG*, and *PN* separately to explore the impact of NUS on SNR, including degrees of 75%, 60%, 50%, 40%, 30%, and 25%. The average SNR of each signal in the six samples was shown in Table S2. It was found that the SNR of small peaks did not present a significant downward trend at 25% of NUS. Accordingly, we preferred to work at 25% of NUS for bs-HSQC.

Above all, we used bs-HSQC combined with 25% NUS to collect spectra of ginsenosides extract with a Bruker AVANCE Ⅲ 500 instrument equipped with a CRYO probe. The Bruker software Topspin 3.6.2 was used. The pulse program of bs-HSQC is shsqcetgisisp 2.2 with Echo/Antiecho-TPPI gradient selection for phase sensitive, shaped pulses for all 180 degree pulses on the f2-channel and gradients in back-inept. The excited carbon region was set between 92 and 112 ppm, with 10 ppm in proton dimension. The coupling constant *^1^J_CH_* was fixed at 145 Hz. 1024 × 128 data points, with eight scans per FID and interscan delay D1 of 2.0 s, resulting in a total acquisition time of 9 min 43 s for each bs-HSQC spectrum. The average SNR of each cross peak obtained by bs-HSQC with 25% NUS was shown in Table 1.

### 2.2. Performance Evaluation

The bs-HSQC coupled with 25% NUS minimized the analysis time to 9 min 43 s, which was validated for precision, repeatability, and the stability of the solution in the following way, and the relative standard deviations (RSDs) were calculated. The precision was assessed by running the same sample six-fold. For repeatability, six samples were prepared, and each sample measured once. Evaluation of solution stability was achieved by collecting bs-HSQC at 0, 2, 4, 8, 12, and 24 h. *PQ*, *PG*, and *PN* samples were all verified as above. Ginsenoside Re, Rf, and R1 had two signals in the excitation region separately. We only selected the integral values of *δ* 101.75/6.5 (Re-1‴), *δ* 103.55/5.93 (Rf-1″), and *δ* 104.55/5.78 (R1-1‴) for analysis. The results showed that the RSD for most peaks was less than 10%, except Rc-1″, Unknown1 and 2 (Table 2).

### 2.3. Species Distinguish

**Figure 3:**
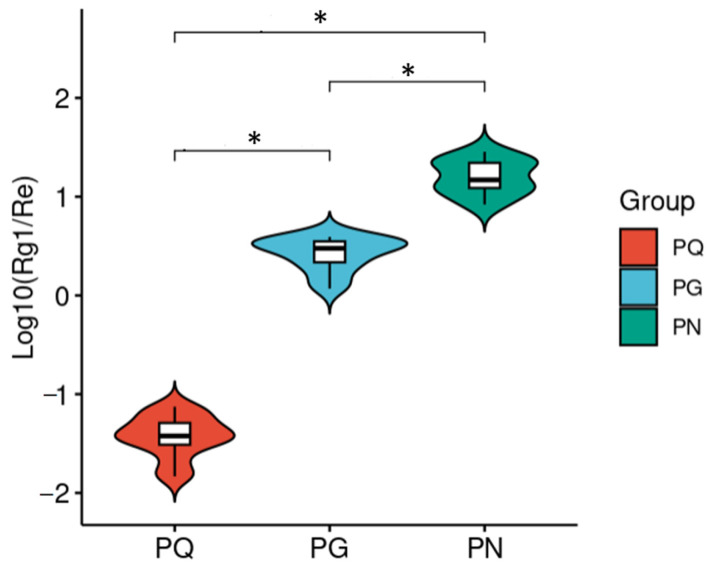
The integral ratio of ginsenoside Rg1 to Re (Rg1/Re) was significantly different between any two groups. “*”, *p* < 0.01.

According to a previous report [10], the ratio of ginsenoside Rg1 to Re in *PQ*, *PG*, and *PN* varies, which can be regarded as an index to distinguish the species. Our work showed that the integral ratio of ginsenoside Rg1 to Re (Rg1/Re) was obviously different (Figure 3; the integral ratio was in Table S3). The *t*-test revealed that the *p*-values of log10(Rg1/Re) between any two groupswere all significant (*p*-values < 0.01). Therefore, the integral ratio of Rg1 to Re can also serve as an index to distinguish *PQ*, *PG*, and *PN*.

**Figure 4:**
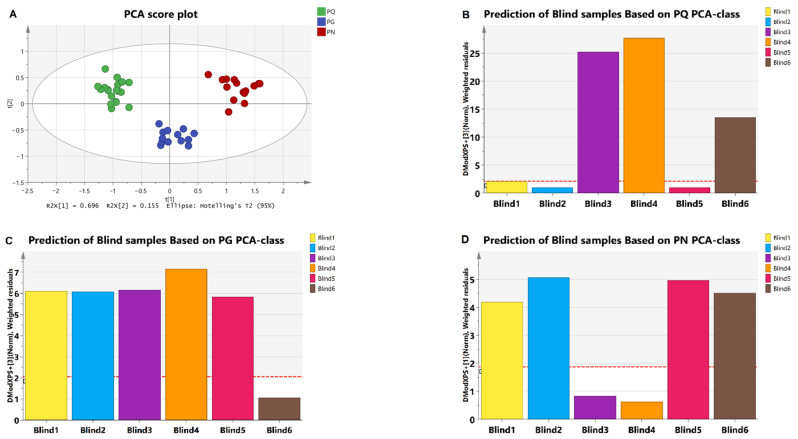
(**A**) The PCA score plot of *PQ*, *PG*, and *PN* samples. The color green represents *PQ*, blue represents *PG*, and red represents *PN*. (**B**) The prediction of blind samples based on *PQ* PCA-class. The blind samples 1, 2, and 5 were lower than Dcrit. (**C**) The prediction of blind samples based on *PG* PCA-class. The blind sample 6 was lower than Dcrit. (**D**) The prediction of blind samples based on *PN* PCA-class. The blind samples 3 and 4 were lower than Dcrit. The red dotted line indicates Dcrit at 95% significance level. When the Mahalanobis distance for a certain sample was lower than Dcrit of that model, the sample was considered positive. Colors represent different samples. The horizontal axis represents the sample name.

After excluding Rc-1″, Unknown1, and Unknown 2 due to large RSD (Table 2), we used eight integral values as variables for multivariate statistics analysis. First, we performed PCA on known samples, and the results showed that all samples appeared within the 95% confidence interval with good clustering, and clear separation among groups (R^2^X = 0.98, Q^2^ = 0.87, Figure 4A). It suggested that the model was stable, and variables were well explained. Therefore, the integration was used to establish PCA-class model to predict the blind samples. The samples were identified by Mahalanobis distance (DModX PS+). When the Mahalanobis distance for a certain sample was lower than Dcrit of that model, the sample was considered to be positive [31]. The results showed that the blind 1, 2, and 5 were *PQ*, 6 was *PG*, and 3 and 4 were *PN* (Figure 4B–D). The sampler (Haowen Zhu) confirmed that all these blind samples’ predictions were correct.

### 2.4. Identification of Adulteration

The results confirmed that the signals in the excited region can accurately reflect the species of the sample, so the established models were used to identify adulterated samples. As saponin extract and herbs in the form of powders and slices are the main commodity, they are often subject to adulteration. Therefore, the study focuses on identifying adulteration at extract and herb levels. Since preparing a large number of adulterated samples is expensive, and the mode of adulteration is comparable between signal mixing and extract mixing, we simulated adulteration at the signal level to observe the identification trend for adulteration behaviors.

#### 2.4.1. Simulative Adulterated Samples at the Signal Mixing Level

**Figure 5:**
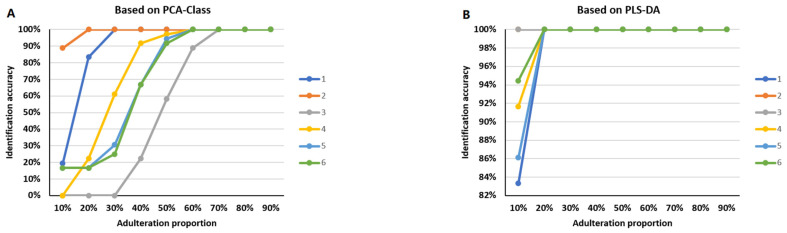
The results of identification accuracy for simulative adulterated samples at the mixing signal level. The colors represent different adulterated samples. The horizontal axis represents the proportion of adulterants. The vertical axis represents the identification accuracy. (**A**) The identification accuracy based on PCA-class. (**B**) The identification accuracy based on PLS-DA, which had better results with higher identification accuracy for low-adulteration ratio than PCA-class. For both PCA-class and PLS-DA, the accuracy of identification increased as the proportion of adulterant increased. 1: The identification accuracy of *PQ* adulteration mixed with *PG* in 9 proportions; 2: The identification accuracy of *PQ* adulteration mixed with *PN* in 9 proportions; 3: The identification accuracy of *PG* adulteration mixed with *PQ* in 9 proportions; 4: The identification accuracy of *PG* adulteration mixed with *PN* in 9 proportions; 5: The identification accuracy of *PN* adulteration mixed with *PQ* in 9 proportions; 6: The identification accuracy of *PN* adulteration mixed with *PG* in 9 proportions.

We prepared 324 simulative adulterated samples for each medicinal material through signal mixing, with a total of nine proportions and 36 samples for each proportion (Table S5). The results showed that the identification accuracy was greater than 80% when the signal of *PQ* was mixed with 20% *PG* (line 1) or 10% *PN* (line 2). The accuracy was 58% and 91% when *PG* was mixed with 50% *PQ* (line 3) or 40% *PN* (line 4), respectively. The accuracy was higher than 90% when *PN* was mixed with 50% *PQ* (line 5) or *PG* (line 6) (Figure 5A; the specific accuracy was shown in Table S4). Notably, if a PLS-DA model was used instead of PCA-class, the identification accuracy achieved 100% for all binary mixtures when the adulteration proportion was 20% and above (Figure 5B).

#### 2.4.2. Adulterated Samples at the Saponins Extract Level

**Figure 6:**
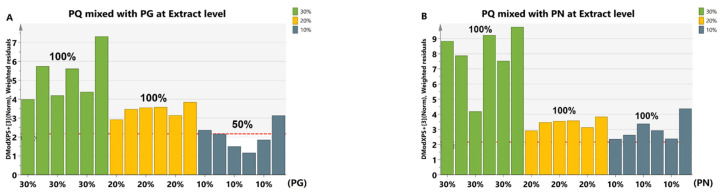
The identification results of *PQ* adulteration at the extract level based on the *PQ* PCA-class model. The red dotted line indicates Dcrit at 95% significance level. When the Mahalanobis distance for a certain sample was lower than Dcrit of that model, the sample was considered positive. Colors represent adulterated samples mixed with different proportions of adulterant. The horizontal axis represents the adulteration proportion. The percentage on the column represents the identification accuracy. (**A**) The identification results of *PQ* ginsenosides extract mixed with that of *PG* in three proportions. There were six samples for each proportion. Three 90% *PQ* + 10% *PG* samples were lower than Dcrit, and other samples were higher than Dcrit. (**B**) The identification results of *PQ* ginsenosides extract mixed with that of *PN* in three proportions. There were six samples for each proportion. All samples were higher than Dcrit.

The noise and signal drift during sample collection may cause the mixed signal to be unable to completely reflect the signal of the actual adulteration at extract level. So, we took actual adulterated *PQ* extract for verification of the developed method. Two types of adulterated samples were prepared by mixing *PQ* with *PG* or *PN*. The proportions of adulterant were 10%, 20%, and 30%, with six samples for each proportion. A total of 36 samples were analyzed (Table S6). The results showed that the identification accuracy was 100% when 20% *PG* (Figure 6A) or 10% *PN* (Figure 6B) were added separately, whose identification trend was basically consistent with the discrimination of simulative adulterated samples (Lines 1, 2 in Figure 5A).

#### 2.4.3. Adulterated Samples at the Herb Level

**Figure 7:**
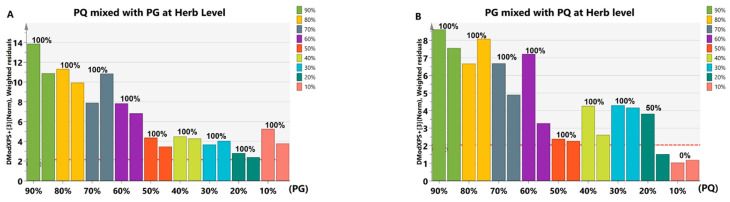
The identification results of adulterated samples at the herb level. (**A**) The identification results of *PQ* mixed with *PG* in 9 proportions. There were two samples for each proportion. All samples were found to be higher than Dcrit. (**B**) The identification results of *PG* mixed with *PQ* in 9 proportions. There were two samples for each proportion. Two 90% *PG* + 10% *PQ* and one 80% *PG* + 20% *PQ* samples were lower than Dcrit. Other samples were found to be higher than Dcrit. The red dotted line indicates Dcrit at 95% significance level. When the Mahalanobis distance for a certain sample was lower than Dcrit of that model, the sample was considered positive. Different colors represent adulterated samples mixed with different proportion of adulterant. The horizontal axis represents the adulteration proportion. The percentage on the column represents the identification accuracy.

As the appearance and slice of *PQ* and *PG* are highly similar, we focused on their adulteration with each other at the herb level. We obtained 18 samples by mixing *PQ* and *PG* in nine proportions with adulteration proportions ranging from 10% to 90%, with 2 samples for each proportion (Table S7). The results showed that they could be identified when *PQ* was mixed with *PG* (at all studied adulteration proportions) (Figure 7A) and when *PG* was mixed with at least 20% *PQ* (Figure 7B). Based on the result, the number of 90% *PQ* + 10% *PG* and 80% *PG* + 20% *PQ* samples increased to six, and the accuracy was found to be 100% and 50%, respectively.

**Figure 8:**
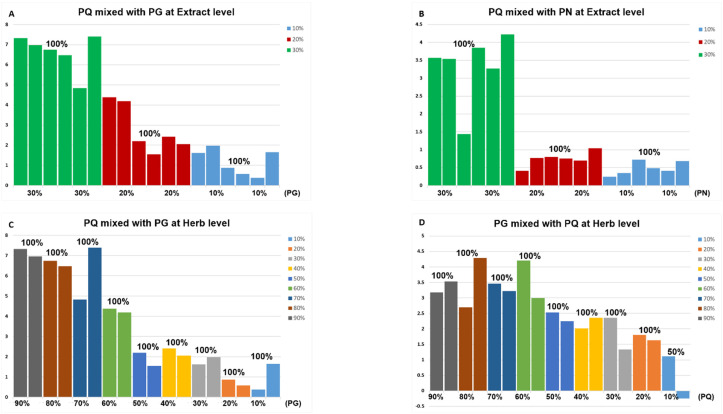
The identification results of adulterated samples based on the corresponding PLS-DA model. (**A**) *PQ* ginsenosides extract mixed with that of *PG* in 3 proportions. There were two samples for each proportion, and all columns were greater than 0. (**B**) *PQ* ginsenosides extract mixed with that of *PN* in three proportions. There were six samples for each proportion, and all columns were greater than 0. (**C**) *PQ* herb mixed with *PG* in nine proportions. There were two samples for each proportion, and all columns were greater than 0. (**D**) *PG* herb mixed with *PQ* in nine proportions. There were two samples for each proportion. One column for 90% *PG* + 10% *PQ* was lower than 0, which means that the result was negative, and other columns were greater than 0. When the column for a certain sample was greater than 0, the sample was considered positive (adulterated samples). Different colors represent adulterated samples mixed with different proportion of adulterant. The horizontal axis represents the adulteration proportion. The percentage on the column represents the identification accuracy.

As the identification results based on PCA-class model were less ideal for some adulteration behaviors, the data were further analyzed using the PLS-DA. The PLS-DA score plots comparing pure samples with 10% adulterated samples were shown in Figure S2, and the PLS-DA models were further used to identify adulterated samples. The detection accuracy of adulteration increased with the increased percentage of adulterant for both PCA-class and PLS-DA, and PLS-DA had better results with higher identification accuracy for low-adulteration ratio than the PCA-class model. Specifically, in the simulative adulterated samples, it appeared that *PN* adulteration was not accurately distinguished at levels below 40%, and it was even worse when *PG* was mixed with *PQ* on the basis of PCA-class model. The situation became better when PLS-DA was adopted (lines 1–6 in Figure 4B and Figure S3). For the *PQ* adulteration at extract level, the detection accuracy of 10% adulteration ratio was 100% (Figure 8A,B), and for adulterated samples at the herb level, the 90% *PQ* + 10% *PG* and 90% *PG +* 10% *PQ* can be identified (Figure 8C,D). Thus, the number of the two samples was increased to six for further identification, and the accuracy for both was found to be 83.33% (the specific accuracy is shown in Table S4).

## 3. Materials and Methods

### 3.1. Materials

#### 3.1.1. Samples Collection

A total of 49 samples were collected and identified by three experts from the Institute of Meteria Medica, Chinese Academy of Medical Sciences and Peking Union Medical College. The certified samples included 19 *PQ*, 14 *PG*, and 15 *PN* standards, with various sources and specifications (Table S3). Additionally, six samples purchased from the market with non-specified species were included.

#### 3.1.2. Extraction of Saponins

A total of 10 g of ground sample was added to a 100 mL round-bottom flask. The sample was extracted under reflux sequentially for 1.5 h with two aliquots (40 mL, 40 mL) of 95% ethanol, followed by vacuum filtration and water bath evaporation of ethanol. Next, the sample was added to 10 mL distilled water, transferred to a separatory funnel, extracted with the same volume of ethyl acetate three times, and then extracted with 15 mL butanol twice. The butanol layers were combined and extracted with 15 mL 2.5% Na_2_CO_3_ three times and extracted with 15 mL distilled water twice. Finally, butanol was evaporated in a water bath. For more details, refer to [32].

#### 3.1.3. Adulterated Samples Preparation

The adulterated samples were prepared from three aspects: digitally diluted adulterated data (mixing of NMR signals), physically diluted adulterated extract, and physically diluted herb.

(1) Digitally diluted adulterated data: We took 6 groups of NMR signals from *PQ, PG*, and *PN* separately. The integration was multiplied by scale factors according to the desired relative content of saponins extract, and then added the obtained data correspondingly to generate simulative adulterated samples at the signal mixing level. For example, in order to obtain the samples of *PQ* mixed with 10% *PG*, the integration from pure *PQ* and *PG* was multiplied by 90% and 10%, respectively. Then, the obtained values were added correspondingly to obtain the theoretical integration of 90% *PQ* + 10% *PG*. In this way, we obtained *PQ* adulteration mixed with *PG* and *PN*, respectively. The proportions of adulterant were 10%, 20%, 30%, 40%, 50%, 60%, 70%, 80%, and 90%. There were 36 samples for each proportion (Table S5). *PG* and *PN* adulteration was obtained in the same way;
(2) Physically diluted adulterated extract: We took 4 samples of saponins extract of *PQ*, *PG*, and *PN* separately. They were prepared in 10 mg/mL mother liquor with Pyr-d5, respectively. Then, according to the desired relative content, we took the corresponding volume of mother liquor and mixed it to obtain adulterated samples at the saponins extract level. For example, in order to obtain the *PQ* extracts mixed with that of 10% *PG*, 720 μL *PQ* extract solution and 80 μL *PG* were mixed to obtain 90% *PQ* + 10% *PG*. In this way, we obtained the *PQ* adulteration at extract level mixed with *PG* in 3 proportions of 10%, 20%, and 30%, respectively. There were 6 samples for each proportion. A total of 18 samples were obtained (Table S6). The *PQ* adulteration mixed with *PN* at extract level was obtained in the same way. The proportions of *PN* were 10%, 20%, and 30%, respectively. A total of 18 samples were obtained;
(3) Physically diluted adulterated herb: Take 1 sample of *PQ* and 2 of *PG*. They were crushed and mixed in a glass bottle according to the desired relative mass ratio to obtain adulterated samples at the herb level. For example, to obtain a sample of *PQ* mixed with 10% *PG*, 9 g *PQ* and 1 g *PG* were mixed. In this way, we obtained the mixtures of *PQ* mixed with *PG* in 9 proportions of 10%, 20%, 30%, 40%, 50%, 60%, 70%, 80%, and 90%, respectively. A total of 18 samples were obtained with 2 in each proportion (Table S7).

### 3.2. Methods

#### 3.2.1. NMR Sample Preparation

For NMR analysis, ginsenosides extract was dissolved with Pyr-d5 to be made into 10 mg/mL with 0.025 mg/mL phloroglucin as an internal standard (IS). After sonication, 600 μL of solution was transferred to a 5 mm NMR tube.

#### 3.2.2. Data Processing

Bs-HSQC spectra were processed using MestReNova x64 version 14.2.1 software. After Fourier transformation of each FID, the spectra were subjected to phase correction, baseline calibration, and integration. All detected peaks were integrated using the elliptical area automatically, utilizing phloroglucinol as IS for calibration.

Using a rectangular area composed of 108–110 ppm in the f1 domain and 7–8 ppm in the f2 as noise, the SNR for each peak was calculated.

#### 3.2.3. Multivariate Statistical Analysis

The data were analyzed using multivariate statistical analysis (SIMCA V14.1, Umetrics, Umea, Sweden). Data were mean-centered and scaled using the Pareto method [33]. Principal component analysis (PCA) was conducted for sample clustering and classification. PCA-class and Mahalanobis distance (DModX PS+) were applied for modeling and prediction, respectively.

In addition, a partial least squares–discriminant analysis (PLS–DA) model was established by using 10% adulterated and pure samples as positive and negative samples, respectively. Considering the imbalance of the number of the two samples, we made one copy of the negative sample to form a new dataset with the positive samples, which were normalized by Pareto scaling and then analyzed by PLS-DA.

## 4. Conclusions

The present work utilized 2D bs-HSQC NMR in combination with NUS and multivariate statistical analysis to establish a method for identifying species and mutual adulteration of *Panax Linn*.

The obtained PCA score plot with distinct separation among *PQ*, *PG*, and *PN* groups demonstrated that the adopted integral value, as a variable, was significant. The subsequent PCA-class model successfully identified blind samples. In addition, our finding was similar with the previous research based on HPLC [10], indicating that the integral ratio of ginsenoside Rg1 to Re obtained based on bs-HSQC spectra can be used as a maker to distinguish the species. For the identification of adulteration, PLS-DA provided better results and had a high detection rate for low-proportion adulteration, with an identification accuracy of greater than 80% when the proportion of adulterants was 10%.

Bs-HSQC NMR can obtain high-resolution spectra in a short time, and it is convenient to operate. First, HSQC uses a wider indirect dimension to achieve separation of overlapped peaks in ^1^H NMR. Then, compared with typical HSQC, bs-HSQC with 25% of NUS can reduce the number of FID, minimizing analysis time while maintaining high-quality data in terms of repeatability and precision. Moreover, using multivariate statistical analysis for relative quantitative analysis can effectively avoid the defects of absolute quantification. It is unnecessary to consider *T1*, heteronuclear coupling constant (*J_CH_*), *J_HHi_*, and other factors affecting integral intensity [20]. Consequently, the collection time was greatly reduced, and the experimental process was brief.

In order to further improve the reliability for the prediction of blind and adulterated samples, the established model may be expanded by introducing more sample sizes from diverse sources. Furthermore, we can develop prediction models for the origin, harvest time, and growth years of *PQ*, *PG*, and *PN*, which would further enhance the 2D NMR-based technology platform for quality control of *Panax Linn*. This would have significant implications for the production, processing, and new drug development of *Panax Linn*, contributing to the assurance of product quality. Given the notable advantages of 2D bs-HSQC NMR, the method may also be extended to the quality monitoring of other traditional Chinese medicines, foods, and agricultural products.

## Figures and Tables

**Table 1:** The resonance assignment and the average SNR of each cross peak in bs-HSQC with 25% NUS (500 MHz, Pyr-d5).

No.	*δ_C_*	*δ_H_*	Attribution	*PQ*	*PG*	*PN*	Statistical Variable
1	101.75	6.50	Re-1‴	248.78	63.29	31.15	Y
2	103.55	5.93	Rf-1″	-	23.71	-	Y
3	104.55	5.78	R1-1‴	-	19.70	51.10	Y
4	109.94	5.66	Rc-1″	17.37	31.04	-	N
5	105.79	5.37	Rc-1′′′′ + Rb1-1′′′′	140.38	146.42	120.68	Y
6	101.64	5.25	Re-1″	163.80	39.03	16.64	N
7	97.99	5.14	^1^H/^13^C-1′	265.80	260.36	332.82	Y
8	105.16	5.09	Rb1-1″	118.73	75.64	91.10	Y
9	105.72	5.01	Rg1-1″	15.98	116.42	248.25	Y
10	104.52	4.99	Unknown1	-	16.45	-	N
11	106.78	4.93	Unknown2	21.38	-	-	N
12	103.33	4.91	R1-1″	-	-	29.71	N
13	103.66	4.91	Rf-1′	-	13.43	-	N
14	104.89	4.90	Rd-1″ +Rc-1‴ +Rb1-1‴	128.07	118.61	106.73	Y

Note: “-” indicates that there was no corresponding ginsenoside; “Y” indicates that the integral value of the corresponding cross peak was used as a variable for multivariate statistical analysis; and “N” indicates it was not used as a variable.

**Table 2:** The relative standard deviations (RSDs) for evaluation of signal intensity of anomeric cross-peaks in the bs-HSQC NMR spectra with 25% NUS (500 MHz, Pyr-d5).

Ginsenosides	Precision (%)			Repeatability (%)			Stability (%)		
	*PQ*	*PG*	*PN*	*PQ*	*PG*	*PN*	*PQ*	*PG*	*PN*
Re-1‴	3.84	12.99	23.77	1.63	6.36	8.21	0.85	7.28	22.27
Rf-1″	-	11.92	-	-	17.89	-	-	14.57	-
R1-1‴	-	-	7.77	-	-	7.40	-	0.00	0.00
Rc-1″	22.27	8.45	-	33.47	12.65	-	10.65	0.00	-
Rc-1′′′′ + Rb1-1′′′′	5.73	1.45	6.51	1.86	4.13	3.74	2.26	1.70	1.68
^1^H/^13^C-1′	3.00	3.26	5.03	1.59	2.39	2.84	1.09	6.64	0.83
Rb1-1″	1.86	1.50	9.25	1.60	7.97	5.32	2.67	11.89	1.93
Rg1-1″	-	3.19	9.32	22.27	2.53	3.32	14.41	2.35	0.85
Unknown1	-	0.00	-	-	31.05	-	-	22.13	-
Unknown2	16.85	-	-	9.96	-	-	41.06	-	24.49
Rd-1″ +Rc-1‴ +Rb1-1‴	1.90	1.64	8.66	0.71	4.47	5.12	1.03	2.07	3.96
Average	6.45			5.57			4.78		

Note: “-”, the medicinal materials do not contain this signal. The average value in the last row was calculated by excluding Rc-1″, Unknown1, and Unknown2 with large RSD.

## Data Availability

The data presented in this study are available on request.

## Supplementary Materials

The following supporting information can be downloaded at: https://www.mdpi.com/article/10.3390/molecules28114332/s1, Figure S1: The identification results of *PQ*, *PG* and *PN* adulteration at signal mixing level based on the corresponding PCA-class model; Figure S2: The PLS-DA score plots of 10% adulterated and pure samples; Figure S3: The identification results of *PQ*, *PG* and *PN* adulteration at signal mixing level based on the corresponding PLS-DA model; Figure S4: Samples display. (A) *PQ*; (B) *PG*; (C) *PN*; Table S1: The average SNR from six samples of each species with different NS for the bs-HSQC NMR; Table S2: The average SNR from six samples of each species with different NUS; Table S3: Sample information and the integral ratio of ginsenoside Rg1 to Re; Table S4: Identification accuracy of adulteration by PCA-class and PLS-DA; Table S5: Calculation methods for obtaining mixed signal of cross peaks in bs-HSQC with 25% NUS; Table S6: Methods for obtaining adulterated samples at the saponins extract level; Table S7: Methods of obtaining adulterated samples at the herb level.
