# Supplementary material for: Application of Band-Selective HSQC NMR in Species Discrimination and Adulteration Identification of Panax Linn
Source: Molecules. 2023 May 25;28(11):4332. doi: 10.3390/molecules28114332 (PMC10254554; doi:10.3390/molecules28114332)
Supplement: Supplementary file 1 [file molecules-28-04332-s001.zip › Supplementary infromation.pdf]

Supporting Material for Application of Band-Selective HSQC NMR in Species discriminant and Adulteration identification of *Panax Linn*

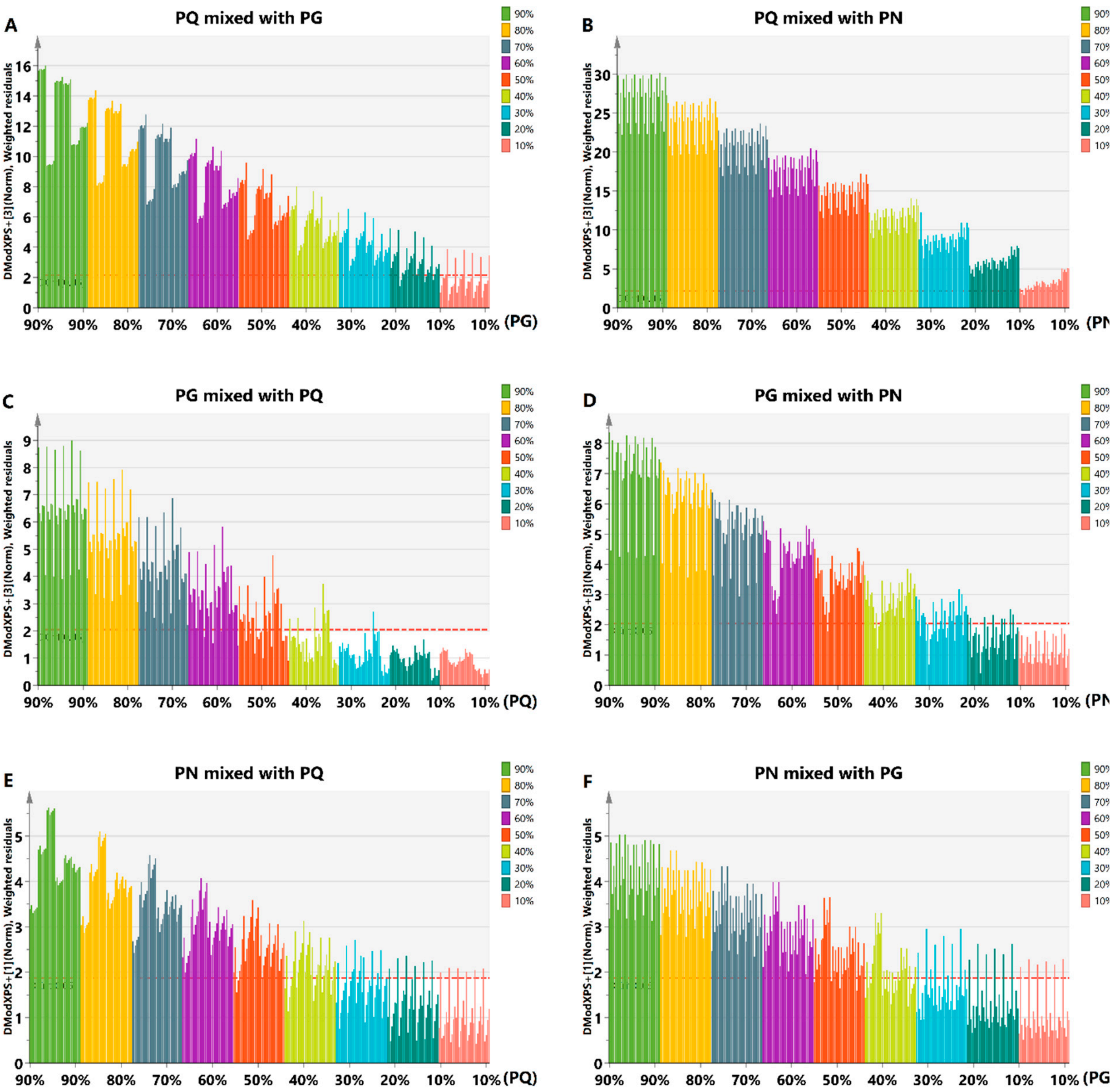

Figure S1. The identification results of *PQ*, *PG* and *PN* adulteration at signal mixing level based on the corresponding PCA-class model. The red dotted line indicated Dcrit at 95% significance level. Colors represented adulterated samples with different proportion of adulterant. The horizontal axis represented the adulteration proportion. **(A):** Identification results of *PQ* mixed with *PG* based on *PQ* PCA-class. When the adulteration ratio was 10%, the sample began to be

higher than Dcrit. **(B):** Identification results of *PQ* mixed with *PN* based on *PQ* PCA-class. When the adulteration ratio was 10%, the sample began to be higher than Dcrit. **(C):** Identification results of *PG* mixed with *PQ* based on *PG* PCA-class. When the adulteration ratio was 50%, the samples began to be higher than Dcrit. **(D):** Identification results of *PG* mixed with *PN* based on *PG* PCA-class. When the adulteration ratio was 30%, more sample began to be higher than Dcrit. **(E):** Identification results of *PN* mixed with *PQ* based on *PN* PCA-class. When the adulteration ratio was 40%, more sample began to be higher than Dcrit. **(F):** Identification results of *PN* mixed with *PG* based on *PN* PCA-class. When the adulteration ratio was 40%, more sample began to be higher than Dcrit.

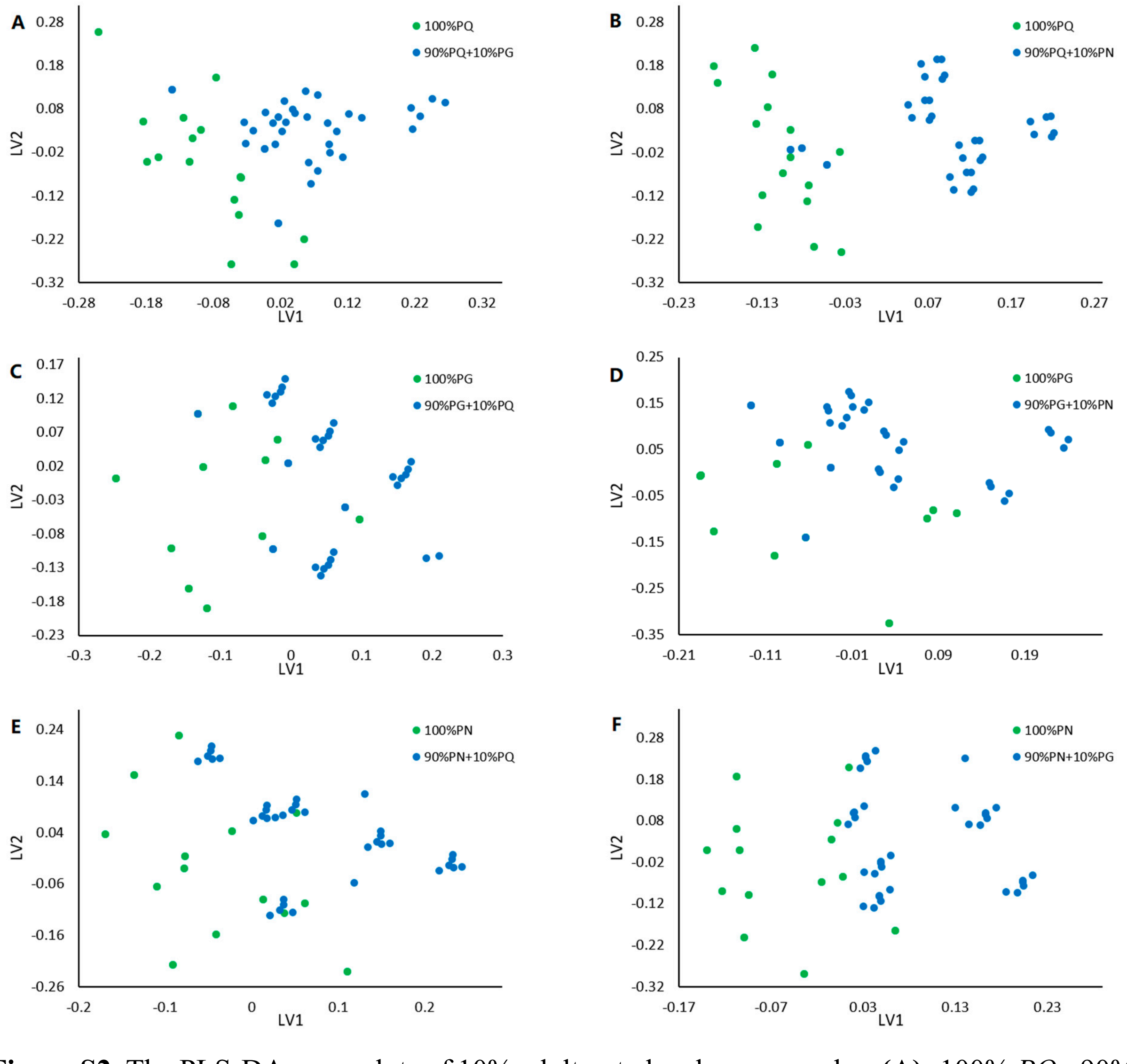

Figure S2. The PLS-DA score plots of 10% adulterated and pure samples. **(A):** 100% *PQ* - 90% *PQ* + 10% *PG*; **(B):** 100% *PQ* - 90% *PQ* + 10% *PN*; **(C):** 100% *PG* - 90% *PG* + 10% *PQ*; **(D):**

100% *PG* - 90% *PG* + 10% *PN*; **(E):** 100% *PN* - 90% *PN* + 10% *PG*; **(F):** 100% *PN* - 90% *PN* + 10% *PQ*.

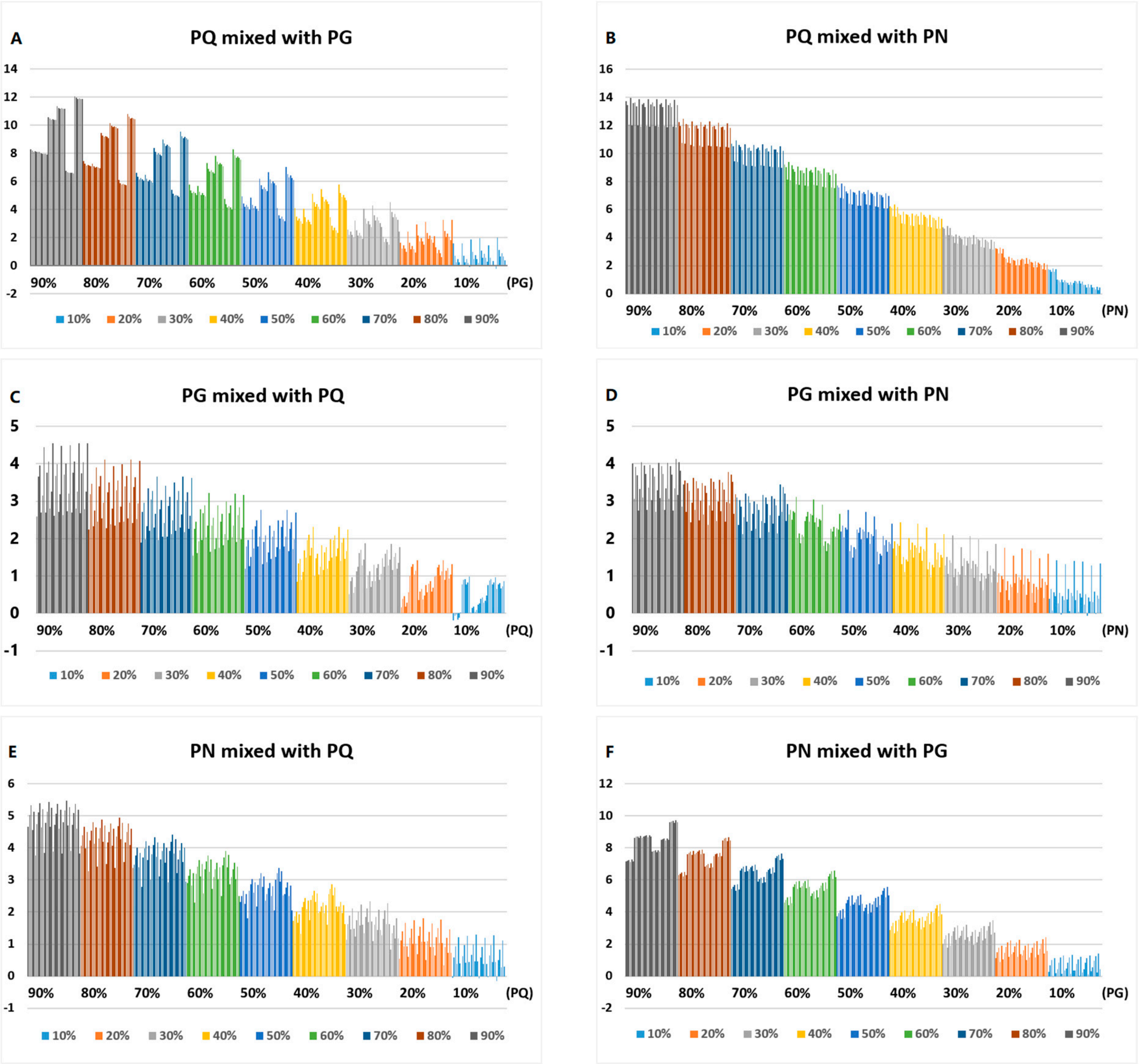

Figure S3. The identification results of *PQ*, *PG* and *PN* adulteration at signal mixing level based on the corresponding PLS-DA model. When the column for a certain sample was greater than 0, the sample was considered positive (adulterated samples). Different color represented adulterated samples mixed with different proportion of adulterant. The horizontal axis represented the adulteration proportion. The column for most adulterated samples were greater than 0, except for a few samples with the adulteration proportion of 10%. **(A):** Identification results of *PQ* mixed with *PG*; **(B):** Identification results of *PQ* mixed with *PN*; **(C):** Identification results of *PG* mixed with *PQ*; **(D):** Identification results of *PG* mixed with *PN*; **(E):** Identification results of *PN* mixed with *PQ*. **(F):** Identification results of *PN* mixed with

PG.

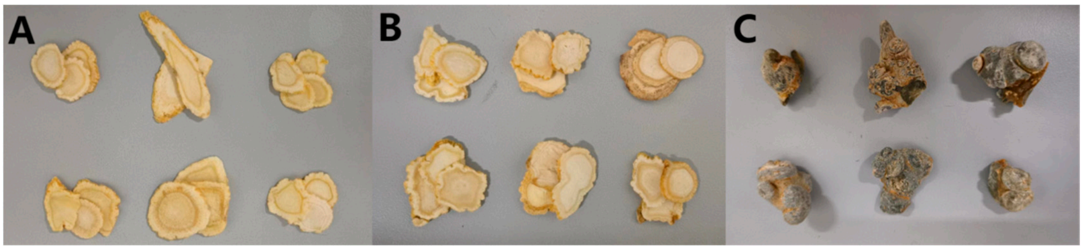

Figure S4. Samples display. **(A):** *PQ*; **(B):** *PG*; **(C):** *PN*.

Table S1. The average SNR from six samples of each species with different NS for the bs-HSQC NMR. The red font is emphasized in the report.

NS		Attribution	4			8			12			16		
**No.**	**f1/f2 (ppm)**		***PQ***	***PG***	***PN***	***PQ***	***PG***	***PN***	***PQ***	***PG***	***PN***	***PQ***	***PG***	***PN***
1	101.75/6.50	Re-1‴	133.96	31.85	17.31	199.24	46.08	23.94	228.34	57.21	28.94	264.59	65.25	34.20
2	103.55/5.93	Rf-1″	-	15.27	-	-	21.22	-	-	25.89	-	-	30.69	-
3	104.55/5.78	R1-1‴	-	7.93	28.35	-	10.65	38.40	-	14.20	47.15	-	19.41	55.35
4	109.94/5.66	Rc-1″	10.83	15.84	-	13.79	22.29	-	17.38	28.41	-	20.59	32.22	-
5	105.79/5.37	Rc-1⁗ + Rb1-1⁗	76.54	72.69	63.02	104.53	104.24	87.45	130.24	127.51	104.89	150.19	148.00	124.62
6	101.64/5.25	Re-1″	91.63	22.23	14.56	129.12	31.31	16.67	156.91	39.84	19.80	181.73	44.90	23.22
7	97.99/5.14	^{1}H-^{13}C-1′	144.65	128.08	182.68	202.47	177.73	246.41	244.27	227.02	297.05	284.04	260.56	344.79
8	105.16/5.09	Rb1-1″	67.69	40.80	53.88	93.27	58.75	73.22	115.77	72.44	86.84	133.78	82.64	101.92
9	105.72/5.01	Rg1-1″	12.26	57.87	130.98	16.50	82.40	180.85	20.13	102.62	218.32	23.96	117.42	257.33
10	104.52/4.99	Unknown1	-	10.47	-	-	15.22	-	-	19.02	-	-	21.83	-
11	106.78/4.93	Unknown2	10.40	-	-	14.31	-	-	17.38	-	-	20.24	-	-
12	103.33/4.91	R1-1″	-	-	18.93	-	-	26.35	-	-	31.77	-	-	37.95
13	103.66/4.91	Rf-1′	-	10.44	-	-	15.11	-	-	18.02	-	-	21.63	-
14	104.89/4.90	Rd-1″ +Rc-1‴ +Rb1-1‴	71.33	62.41	59.30	98.55	88.92	82.91	121.22	112.02	99.81	139.80	126.81	117.82

Table S2. The average SNR from six samples of each species with different NUS. The red font is emphasized in the report.

NUS/%	Attribution	75			60			50			40			30			25		
No.		*PQ*	*PG*	*PN*	*PQ*	*PG*	*PN*	*PQ*	*PG*	*PN*	*PQ*	*PG*	*PN*	*PQ*	*PG*	*PN*	*PQ*	*PG*	*PN*
1	Re-1‴	213.51	51.56	26.27	216.17	52.20	26.29	224.26	54.61	28.05	231.80	58.33	28.23	252.24	60.40	30.75	248.78	63.29	31.15
2	Rf-1″	-	22.40	-	-	23.20	-	-	24.04	-	-	22.54	-	-	24.30	-	-	23.71	-
3	R1-1‴	-	13.59	43.11	-	12.12	41.97	-	12.76	46.41	-	16.56	46.04	-	16.05	50.52	-	19.70	51.10
4	Rc-1″	16.03	25.34	-	16.22	25.21	-	16.79	26.63	-	17.46	28.54	-	19.06	28.51	-	17.37	31.04	-
5	Rc-1⁗ + Rb1-1⁗	120.72	115.56	98.86	123.46	117.27	96.11	124.90	122.80	104.30	131.36	131.43	108.53	141.35	135.92	120.46	140.38	146.42	120.68
6	Re-1″	144.65	34.87	17.12	147.27	35.05	16.75	150.78	36.82	19.48	154.71	37.66	18.74	166.55	36.31	18.68	163.80	39.03	16.64
7	$^{1}H/^{13}C$-1′	228.45	197.81	276.23	235.52	211.27	268.94	234.62	231.43	295.70	244.58	210.57	312.53	273.33	230.61	334.48	265.80	260.36	332.82
8	Rb1-1″	107.29	65.89	78.43	108.27	65.39	77.29	111.93	68.79	84.87	113.80	73.30	83.33	121.81	73.23	94.58	118.73	75.64	91.10
9	Rg1-1″	17.53	93.34	204.47	17.86	94.07	197.90	18.76	97.89	219.38	18.15	105.20	222.01	18.01	107.11	244.89	15.98	116.42	248.25
10	Unknown1	-	16.41	-	-	16.13	-	-	17.20	-	-	18.10	-	-	17.20	-	-	16.45	-
11	Unknown2	15.13	-	-	15.33	-	-	15.42	-	-	17.87	-	-	21.07	-	-	21.38	-	-
12	R1-1″	-	-	28.56	-	-	28.20	-	-	31.25	-	-	27.89	-	-	30.50	-	-	29.71
13	Rf-1′	-	15.73	-	-	15.90	-	-	16.05	-	-	15.16	-	-	12.37	-	-	13.43	-
14	Rd-1″ +Rc-1‴ +Rb1-1‴	112.69	100.18	93.07	113.84	102.30	90.80	117.32	105.68	96.80	121.65	112.16	99.41	130.01	111.24	109.65	128.07	118.61	106.73

Table S3. Sample information and the integral ratio of ginsenoside Rg1 to Re.

Sample	Number	Source	Specification	Rg1-Re
P. quinquefolium	*PQ1*	Jilin	Tablet	0.02
P. quinquefolium	*PQ2*	Canada	Tablet	0.04
P. quinquefolium	*PQ3*	Beijing	Tablet	0.03
P. quinquefolium	*PQ4*	Beijing	Tablet	0.00
P. quinquefolium	*PQ5*	Canada	Tablet	0.04
P. quinquefolium	*PQ6*	Jilin	Tablet	0.00
P. quinquefolium	*PQ7*	22.0913	Tablet	0.00
P. quinquefolium	*PQ8*	Beijing	Tablet	0.03
P. quinquefolium	*PQ9*	50131	Tablet	0.04
P. quinquefolium	*PQ10*	50871	Tablet	0.01
P. quinquefolium	*PQ11*	21101001	Tablet	0.05
P. quinquefolium	*PQ12*	USA	Tablet	0.05
P. quinquefolium	*PQ13*	USA	Tablet	0.05
P. quinquefolium	*PQ14*	Shanxi	Tablet	0.07
P. quinquefolium	*PQ15*	USA	Tablet	0.03
P. quinquefolium	*PQ16*	-	Tablet	0.06
P. quinquefolium	*PQ17*	Shanxi	Tablet	0.04
P. quinquefolium	*PQ18*	Shanxi	Tablet	0.02
P. quinquefolium	*PQ19*	Jilin	Tablet	0.07
P. ginseng	*PG1*	Jilin	Tablet	2.27
P. ginseng	*PG2*	Jilin	Tablet	3.50
P. ginseng	*PG3*	Jilin	Tablet	3.00
P. ginseng	*PG4*	Jilin	Tablet	3.00
P. ginseng	*PG5*	Jilin	Tablet	1.17
P. ginseng	*PG6*	Jilin	Tablet	1.82
P. ginseng	*PG7*	Liaoning	Tablet	3.90
P. ginseng	*PG8*	Jilin	Tablet	2.79

P. ginseng	*PG9*	Jilin	Tablet	1.38
P. ginseng	*PG10*	Jilin	Tablet	2.13
P. ginseng	*PG11*	Jilin	Tablet	3.92
P. ginseng	*PG12*	Jilin	Tablet	3.42
P. ginseng	*PG13*	Jilin	Tablet	3.54
P. ginseng	*PG14*	Jilin	Tablet	3.56
P. notoginseng	*PN1*	Yunnan	Xiaozhitou	14.80
P. notoginseng	*PN2*	Yunnan	40tou	19.00
P. notoginseng	*PN3*	Yunnan	-	21.79
P. notoginseng	*PN4*	Yunnan	20tou	28.50
P. notoginseng	*PN5*	Yunnan	40tou	8.33
P. notoginseng	*PN6*	Yunnan	80tou	21.67
P. notoginseng	*PN7*	Yunnan	Washing 60tou	13.86
P. notoginseng	*PN8*	Yunnan	Washing 80tou	23.25
P. notoginseng	*PN9*	Yunnan	-	22.25
P. notoginseng	*PN10*	Yunnan	-	8.56
P. notoginseng	*PN11*	Yunnan	Washing 30tou	12.17
P. notoginseng	*PN12*	Yunnan	Block	10.75
P. notoginseng	*PN13*	Yunnan	Washing 40tou	12.33
P. notoginseng	*PN14*	Yunnan	Pollution-free 40tou	12.43
P. notoginseng	*PN15*	Yunnan	-	26.00

Table S4. Identification accuracy of adulteration by PCA-class and PLS-DA.

**PCA-class model**										
**Adulteration**	**Signal**						**Extract**		**Herb**	
	PQ		*PG*		*PN*		*PQ*		*PQ*	*PG*
Additive / Proportion	*PG*	*PN*	*PQ*	*PN*	*PQ*	*PG*	*PG*	*PN*	*PG*	*PQ*
10%	19.44%	88.89%	0.00%	0%	16.67%	16.67%	50.00%	100%	100%	0.00%
20%	83.33%	100%	0.00%	22.22%	16.67%	16.67%	100%	100%	100%	50%
30%	100%	100%	0.00%	61.11%	30.56%	25.00%	100%	100%	100%	100%
40%	100%	100%	22.22%	91.67%	66.67%	66.67%	-	-	100%	100%
50%	100%	100%	58.33%	97.22%	94.44%	91.67%	-	-	100%	100%
60%	100%	100%	88.89%	100%	100%	100%	-	-	100%	100%
70%	100%	100%	100%	100%	100%	100%	-	-	100%	100%
80%	100%	100%	100%	100%	100%	100%	-	-	100%	100%
90%	100%	100%	100%	100%	100%	100%	-	-	100%	100%
**PLS-DA model**										
10%	83.33%	100%	100%	91.67%	86.11%	94.44%	100%	100%	100%	50.00%
20%	100%	100%	100%	100%	100%	96.00%	100%	100%	100%	100%
30%	100%	100%	100%	100%	100%	100%	100%	100%	100%	100%
40%	100%	100%	100%	100%	100%	100%	-	-	100%	100%
50%	100%	100%	100%	100%	100%	100%	-	-	100%	100%
60%	100%	100%	100%	100%	100%	100%	-	-	100%	100%
70%	100%	100%	100%	100%	100%	100%	-	-	100%	100%
80%	100%	100%	100%	100%	100%	100%	-	-	100%	100%
90%	100%	100%	100%	100%	100%	100%	-	-	100%	100%

Note: “-” indicated that there were no samples.

Table S5. Calculation methods for obtaining mixed signal of cross peaks in bs-HSQC with 25% NUS.

	PQ+PG								
	90%+ 10%	80%+ 20%	70%+ 30%	60%+ 40%	50%+ 50%	40%+ 60%	30%+ 70%	20%+ 80%	10%+ 90%
PQ1+PG1	√	√	√	√	√	√	√	√	√
PQ1+PG2	√	√	√	√	√	√	√	√	√
PQ1+PG3	√	√	√	√	√	√	√	√	√
PQ1+PG4	√	√	√	√	√	√	√	√	√
PQ1+PG5	√	√	√	√	√	√	√	√	√
PQ1+PG6	√	√	√	√	√	√	√	√	√

Note: "√", the corresponding samples were obtained.

In this way, we got the mixed signal in 9 proportions between *PQ* and *PG, PQ* and *PN*, *PG* and *PN* separately, as simulative adulterated samples at the signal mixing level. There were 36 samples for each proportion. A total of 324 simulative adulterated samples were obtained for each medicinal material.

Table S6. Methods for obtaining adulterated samples at the saponins extract level.

	PQ+PG		
	90%+10%	80%+20%	70%+30%
PQ1+PG1	√	√	√
PQ2+PG2	√	√	√
PQ3+PG3	√	√	√
PQ4+PG4	√	√	√
PQ5+PG5	√	√	√
PQ6+PG6	√	√	√

Note: *PQ* and *PG* represented mother liquor of pure *PQ* and *PG* extract respectively. "√", the corresponding samples were obtained.

In this way, we got the *PQ* adulteration at extract level mixed with *PG* in 3 proportions of 10%, 20%, and 30%, respectively. There were 6 samples for each proportion. A total of 18 samples were obtained. The *PQ* adulteration mixed with PN

at extract level were obtained in the same way.

Table S7. Methods of obtaining adulterated samples at the herb level.

	PQ+PG								
	90%+ 10%	80%+ 20%	70%+ 30%	60%+ 40%	50%+ 50%	40%+ 60%	30%+ 70%	20%+ 80%	10%+ 90%
PQ1+PG1	√	√	√	√	√	√	√	√	√
PQ2+PG1	√	√	√	√	√	√	√	√	√

Note: *PQ* and *PG* represented corresponding pure samples. "√", the corresponding samples were obtained.
